# Physicochemical and Sensory Evaluation of Spreads Derived from Fruit Processing By-Products

**DOI:** 10.3390/foods14132224

**Published:** 2025-06-24

**Authors:** Chrysanthi Nouska, Liliana Ciurla, Antoanela Patras, Costas G. Biliaderis, Athina Lazaridou

**Affiliations:** 1 Laboratory of Food Chemistry and Biochemistry, Department of Food Science and Technology, School of Agriculture, Aristotle University of Thessaloniki, P.O. Box 235, 54124 Thessaloniki, Greece; biliader@agro.auth.gr; 2 Faculty of Horticulture, “Ion Ionescu de la Brad” Iasi University of Life Sciences, 3 Mihail Sadoveanu, Alley, 700490 Iasi, Romania; liliana.ciurla@iuls.ro (L.C.); antoanela.patras@iuls.ro (A.P.)

**Keywords:** apple pomace, tomato pomace, grape pomace, fruit pomace spreads, dynamic rheological measurements, mechanical spectra, phenolic compounds profile, principal component analysis, spreadability test, sensory analysis

## Abstract

Apple, tomato, and grape pomaces, as well as an apple–grape (1:1) mixed pomace, were employed in the formulation of fruit-based spreads to valorize these underutilized by-products. The influence of pectin addition on the physicochemical and sensory properties of the spreads was also examined. All spread preparations carried the ‘high fiber’ nutrition claim. The apple pomace spread demonstrated the highest total and soluble dietary fiber contents (14.13 and 4.28%, respectively). Colorimetry showed higher L* and a* values for the tomato pomace spreads. Rheometry of the spreads revealed pseudoplastic flow and weak gel-like behavior (G′ > G″); the tomato and grape pomace spreads with pectin exhibited the highest η*, G′, and G″ values. A texture analysis (spreadability test) indicated that pectin addition affected only the mixed pomace spread, resulting in the least spreadable product. Regarding bioactive compounds, the apple pomace had the highest total phenolic content, and the grape pomace exhibited the highest antioxidant activity, both of which were also reflected in their corresponding spreads. A principal component analysis indicated a strong correlation among flavor, mouthfeel, and moisture content, which were negatively correlated with color intensity and spreadability. The apple pomace spread with added pectin was the most widely preferred by consumers due to its appealing mouthfeel, spreadability and flavor.

## 1. Introduction

After fruit juice extraction, the remaining fruit pomaces often include the skin, pulp, and seeds left as insoluble residues. These materials, representing 20–60% of the raw fruit depending on the source, are increasingly recognized for their nutraceutical potential [1]; i.e., fruit pomaces are rich sources of bioactive compounds, particularly polyphenols, which are known for their antioxidant potential and health improving properties [2]. In addition to polyphenols, fruit pomaces are abundant in dietary fibers, minerals, vitamins, prebiotic oligosaccharides, and carotenoids [3,4], making them valuable for use as functional additives in food and medicinal formulations. Furthermore, the use of fruit by-products in functional foods promotes sustainable development and waste recycling, contributing to a greener environment [5].

Currently, the majority of fruit pomaces are either used as animal feed or discarded in landfills due to the high costs associated with drying, transportation, and storage [6]. This practice raises environmental concerns. Moreover, there is a growing consumer demand for healthier and more natural foods, leading to increased interest in repurposing such by-products for use in food formulations. In recent years, fruit pomaces have been incorporated into various food products, such as bakery items and meat products to enhance dietary fiber content, antioxidant potency, water retention, and flavor notes [1,7,8]. Jam is one of the most popular and widely chosen fruit-based products, providing a means of preserving fruit throughout the year. This popularity makes ‘jam-like’ spreads based on fruit pomaces ideal functional food products. In the wine and juice industry, grape pomace is the primary by-product, and its disposal poses a significant challenge [9]. Due to its low economic value, grape pomace is commonly used as animal feed [10], fertilizer, or for alcohol production. Despite being considered a non-valorized material, grape pomace is a rich source of dietary fibers and contains significant amounts of natural bioactive compounds, such as polyphenols, which contribute to its enhanced antioxidant potential [11,12]. Grape pomace can contain up to 85% dietary fibers, depending on the grape variety, while up to 70% of the phenolic compounds of grapes may remain in the pomace following the winemaking process [13,14]. Given the growing interest in healthier foods, utilizing grape pomace for food fortification is crucial not only to reduce waste but also to enhance the nutritional value of food products. Tomato, a widely produced fruit, is consumed both fresh and in processed forms, such as juice, paste, sauce, ketchup, and puree [15]. Actually, processed tomato products account for one-third of the total tomato production, generating significant amounts of tomato pomace, which primarily consists of pulp, peel, and seeds, comprising 5–30% of the raw material [16,17]. Annually, 5.4 to 9.0 million tons of tomato pomace are produced [18], creating a significant waste disposal challenge for the food-processing industry. Nevertheless, tomato pomace is an excellent source of phenolic compounds (flavonoids, phenolic acids) [19] and other important phytochemicals (tocopherols, carotenoids, sterols), contributing to enhanced antioxidant properties [20]. Apple pomace is the primary by-product of apple juice extraction which is commonly used as animal feed [21], and as a source of pectins [22] and phenolic compounds [23]. Apple pomace contains a high concentration of pectin (13–39%) [24], making the addition of gelling agents unnecessary for jelly production. Pectin, which acts mostly as a soluble dietary fiber in food matrices, is commonly used in jams and jellies, particularly when ~80% esterification of the galacturonic acid residues occurs [25]. Commercial pectins are classified as low (<50%) or high (>50%) methoxyl, according to the degree of methyl esterification. Moreover, European regulations [26] require that commercial pectins contain at least 65% α-D-galacturonic acid.

Hydrocolloids, such as pectins, are widely used as thickening, gelling, or stabilizing agents as they are polymers that are soluble in the water and largely modify their physical properties. The concentration, type of hydrocolloid, pH, processing temperature, and food matrix all influence the thickening properties [27,28]. In jams, gelation occurs after heating and upon cooling, with pectin being dissolved in water at temperatures above 50 °C. The non-polar methoxyl groups in the polymeric chains of pectin disrupt the water structure, reducing its entropy. Consequently, in order to lower this thermodynamically unfavorable process the exposure of methoxyl groups to water is reduced, by their aggregation, thereby leading to junction zone formation among pectin chains. The addition of divalent Ca^2+^ can also foster interchain associations involving free carboxylate groups (ionotropic gelation, known as the ‘egg box’ mechanism). Hydrogen bonds between the hydroxyl groups (-OH) of adjacent molecules and hydrophilic carboxylate groups (-COOH) on the chains of pectin are also responsible for the stabilization of junction zones among the chains. Ultimately, a filamentous polymeric structure is formed by the interacting chains (hydrated polymer network structure), yielding the distinctive gel-like texture encountered in fruit jams [29].

The use of fruit pomaces in jam-like spreads presents a promising approach to utilizing these by-products. This not only contributes to the reduction of food waste but also enables the use of these residues as functional constituents, enhancing the antioxidant activity and dietary fiber content of the end products. In this context, the aim of this study was to explore the potential use of undervalued apple, grape, and tomato pomaces as primary ingredients in spread products. To assess the effectiveness of these by-products, individually or in combination, in such food formulations, various quality parameters were evaluated including pH, acidity, moisture content—water activity (a_w_), color, rheological properties, texture, protein content, dietary fiber, total phenolic content, antioxidant activity, and phenolic compounds. Moreover, the impact of added pectin in fruit pomace-based spreads was investigated.

## 2. Materials and Methods

### 2.1. Fruit Pomace Materials

Three different types of pomaces were used for the spread preparations. All of them were obtained from the north-east of Romania as by-products of various processing methods: the tomato pomace was derived from a small-scale tomato juice preparation, the apple pomace also resulted from juice preparation and was provided by a small local company, while the grape pomace was obtained from wine production and supplied by a winemaking company. All pomaces were collected immediately after juice processing and stabilized by drying them at 60 °C to a constant weight in a convective laboratory oven (Biobase BOV-T30C, Jinan, China); this thermal treatment offered a practical balance between drying efficiency and the retention of quality parameters, and the inhibition of microbiological growth. The dried pomaces were ground, sieved (1 mm), and stored in a cold, dry place until used for spread preparation; particle size reduction aimed to facilitate an even hydration and dispersion of the particles in water, the solubilization of water-soluble components, and the subsequent gelation during spread preparation.

A commercial low-methoxyl amidated pectin was purchased from CP Kelco (GENU Pectin LM-101 AS; CP Kelco, Großenbrode, Germany); according to the manufacturer’s specifications, this product had a degree of esterification, 35%, and a degree of amidation, 15%.

### 2.2. Pomace Spread Production

Apple pomace, tomato pomace, grape pomace and a 1:1 mixture of apple and grape pomace in dry form were employed to prepare the pomace spreads. All the ingredients (Table 1) were combined and heated to 85–90 °C under constant stirring until the mixture reached 65 °Brix, as measured by a refractometer, in order to ensure the appropriate sweetness and enhance the gelling capacity of pectin; moreover, such a solid content meets the requirements of the European Council Directive 2001/113/EC concerning the dry matter content in fruit jams (≥60%) [30]. The hot spreads were transferred into sterilized jars with screw caps and stored at room temperature. The levels of pectin and CaCl_2_ used were selected based on the pectin supplier’s guidelines and preliminary experiments, in order to achieve an appropriate gel structure and product consistency. Moreover, the water content was adjusted according to preliminary experiments to ensure a desirable texture and spreadability of the final products. In tomato and grape pomace spreads, citric acid was also added to lower the pH value to 3.5 and reduce the repulsive forces of ionized free carboxylate groups.

### 2.3. pH, a_w_, Total Titratable Acidity and Moisture Content Determination

The pH was determined using a benchtop pH meter (Bante 210 Benchtop pH/mV Meter, Shangai, China).

A water activity meter (Aqualab 3TE, Decagon Devices, Inc., Pullman, WA, USA) was used to measure the water activity of the samples at 20 °C.

For moisture content analysis, 10 g of each sample was placed in a crucible and heated in an oven at 105 °C until constant weight was reached [31]. The moisture content was calculated based on weight loss (drying) using the following equation:

Moisture content (%) = (fresh weight of sample − dry sample weight/fresh sample weight) × 100(1)

To determine the titratable acidity, 1 g of the sample was weighed and diluted with 75 mL of distilled water. The mixture was stirred until homogenization, and NaOH 0.1 M was added as titrant until the pH reached 8.2 [32]. The volume of NaOH added (ml) was converted to percentages of citric acid.

### 2.4. Protein and Total Dietary Fiber Content of Pomaces

Protein content was determined using the Kjeldahl method [33].

Total dietary fiber (TDF) was measured using the Total Dietary Fiber Assay kit from Megazyme (Megazyme International Ireland Ltd., Co., Wicklow, Ireland), which contains a thermostable α-amylase, a protease, and an amyloglucosidase. To separate insoluble and soluble dietary fiber fractions, the Fiberbags system filtration (Gerhardt Analytical Systems, Königswinter, Germany) was used. The procedure followed the AACC method 32-05 [34] and AOAC Method 985.29 [35], as described elsewhere [36,37].

### 2.5. Color Measurements

The color parameters of the pomace spreads were evaluated using a Chromameter (Konica Minolta, CR-400 Series, Tokyo, Japan) by the CIE system (L*, a* and b* values).

### 2.6. Rheometry

A rotational Physica MCR 300 rheometer (Anton Paar GmbH, Graz, Austria) equipped with a Paar Physica circulating bath and a controlled peltier system (TEZ 150P/MCR), which maintained the temperature accuracy within ±0.1 °C, were used to evaluate the rheological behavior of the pomace spreads.

A plate–plate geometry (50 mm diameter) with a 2 mm gap between the two plates was used. Frequency sweep tests were conducted to determine the storage (G′) and loss (G″) moduli, and damping factor (tanδ) at 0.1% strain level (linear viscoelasticity region), over a frequency range of 0.1–100 Hz at 20 °C.

### 2.7. Texture Analysis

The spreadability test was performed using a Texture Analyser TA.XT plus (Stable Micro Systems, Godalming, Surrey, UK) equipped with a TTC Spreadability Rig (HDP/SR*). The ring consisted of a male 45° cone probe and a precisely matched female cone shaped product holder. To the lower cone holder, portions of pomace spreads were placed, and the surface was leveled. The movable male cone penetrated the sample at a speed of 3 mm/s. Firmness was determined by the maximum force of the positive peak (F_max_), while the spreadability work (SW) was calculated as the area under the curve of the positive peak using the Texture Expert software (version 1.22).

### 2.8. Antioxidant Activity and Phenolic Compound Profile

#### 2.8.1. Extraction Procedure

Extraction of phenolics was performed following the procedure described by Bajić et al. [38] with some modifications. Ground pomace or a portion of lyophilized spread was homogenized with solvent (MeOH 50% + 0.3% HCl) at a ratio of 1:6 (*w*/*v*), using a vortex, for 10 s. Subsequently, the samples were sonicated (50 °C, 30 min) using an ultrasonic bath (HBM GL Serie 2.5 Liter Ultrasoon Reiniger, Moordrecht, The Netherlands) and centrifuged for 10 min, at 5000 rpm and 4 °C (Hettich Zentrifugen Mikro 22R centrifuge, Tuttlingen, Germany). The clear supernatant was collected, filtered through a 0.45 µm membrane and then used for the analysis of total phenolic content, antioxidant capacity, and the chromatographic quantification of polyphenols. All extractions were performed in triplicate.

#### 2.8.2. Determination of Total Phenolic Content

The total phenolic content of the pomaces or spreads was determined using the Folin–Ciocalteu method. A 200 µL aliquot of diluted extracts (1:10) was mixed with 6000 µL of distilled water, 500 µL of Folin–Ciocalteu reagent, and 1500 µL of sodium carbonate (20%). After 120 min of incubation in dark conditions, the absorbance of the sample was read at 760 nm, using the Analytic Jena Specord 200 plus UV–Vis double beam spectrophotometer (Analytik Jena GmbH + Co. KG, Jena, Germany). The results for total polyphenols were calculated using a calibration curve of gallic acid and expressed in mg equivalents of gallic acid/100 g sample.

#### 2.8.3. Determination of the Antioxidant Activity

The antioxidant activity was determined using the ABTS assay [39]. The absorbance was read at 734 nm (Specord 200 plus UV–Vis spectrophotometer, Analytik Jena GmbH + Co. KG, Jena, Germany), 120 s after mixing the extract with ABTS•, using 96% ethanol as reference. The results were calculated using a calibration curve and were expressed as trolox equivalents (µmol TE/100 g).

#### 2.8.4. Chromatographic Analysis of Phenolic Compounds

The Waters 2695e Alliance HPLC system, coupled with a 2998 PDA Detector (Waters, Milford, MA, USA), and controlled by the Empower^®^ 3 software. Separation was accomplished on a Waters XBridge C18 column (50 × 4.6 mm, 3.5 µm), kept at 30 °C. The elution was performed in gradient by mixing the mobile phase A (0.1% trifluoroacetic acid in water) and B (0.1% trifluoroacetic acid in acetonitrile) at a flow rate of 0.7 mL/min. The detailed chromatographic conditions have been previously reported [40]. For the quantification of phenolic compounds external standards were used. The results are presented as µg of phenolic compound/g sample.

### 2.9. Sensory Analysis

Twenty trained assessors participated in the sensory evaluation. The age range of the panelists was from 20 to 45 years, and the gender distribution was approximately 40% male and 60% female. All assessors had prior experience in quantitative descriptive sensory analysis and participated in two training sessions of 2 h each, during which they became familiar with the sensory attributes under evaluation and the use of an unstructured 10 cm scale. During testing, attributes such as a distinct apple, tomato, and grape taste and flavor, and the color of the samples were assessed. Mouthfeel perception was evaluated by instructing assessors to rub the sample on the palate with the tongue for approximately 10 s, focusing on smoothness, grittiness, and the overall mouth texture. The degree of spreadability was evaluated by spreading each sample onto a rusk, using a plastic knife, and performing four spreading movements in different directions (left to right, right to left, repeated once more), to standardize the assessment routine. With the increase in score values from ‘1’ to ‘10’, the darkness of the color, the difficulty of spreading the product (spreadability), the sour taste and the intensity of the distinct flavor note and taste of the respective fruit increased, while the perceived mouthfeel on the palate (related to reduced coarseness) improved. Additionally, the sensory analysis of the pomace spreads included an overall acceptability test with 75 untrained consumers, who rated the pomace spreads on a 9-point hedonic scale based on their overall acceptability of the product.

### 2.10. Statistical Analysis

The experimental data were analyzed using Analysis of Variance (ANOVA) with Tukey’s post hoc test, conducted with IBM SPSS statistical software (version 22.0, IBM Corp., Armonk, NY, USA) at a 95% significance level. For each analysis, at least three samples were measured, and the results expressed as means ± standard deviations.

A dendrogram and a heatmap were generated to visualize the relative concentration variations (z-scores) of phenolic compounds. The dendrogram was constructed using Euclidean distance clustering in SPSS, while the heatmap was created using the Tableau Public platform (version 2024.3, Tableau Software, Seattle, WA, USA).

A Principal Component Analysis (PCA) was performed to correlate the results from instrumental and sensory analyses using Minitab software (version 18, Minitab, Inc., State College, PA, USA). Variables were standardized using z-scores to ensure equal weighting.

## 3. Results and Discussion

### 3.1. Physicochemical Properties of Fruit Pomace Spreads

The pH values (Table 2) were similar among the tested samples, as citric acid was used as an acidity regulator for some of the fruit pomace-based spread formulations. The total titratable acidity was lowest for the GRAP sample and highest for the APPC sample among all formulations.

The water activity (a_w_) values of the fruit pomace spreads ranged from 0.88–0.97 (Table 2), indicating that spreads with such high a_w_ values are quite susceptible to microbial spoilage. Grape pomace spreads, which had the lowest a_w_ values, are expected to spoil at a slower rate compared to the other tested samples. Previous studies have also shown variations in a_w_ values among fruit pomace jams, including apple, pineapple, guava, kinnow, and mixed fruit pomace jams [6]. The a_w_ value of fruit preserves depends on the water content and concentration of small molecular weight solutes (e.g., sugars).

In terms of moisture content, the GRAC sample exhibited the lowest value, while the apple and tomato pomace spreads, both with or without the addition of pectin, showed the highest moisture content (Table 2).

The protein content was higher in the case of grape pomace spreads (Table 3). The total dietary fiber (TDF) of the fruit pomace spreads ranged between 9.85 and 14.13%, with the highest content found in apple pomace spreads (with or without the addition of pectin) and the lowest in tomato pomace spreads. Kapoor et al. [6] tested various fruit pomace jams and found that jams made with fruit pomaces exhibited a higher dietary fiber content compared to commercial jams. According to the EU regulation no. 1924/2006 [41], products containing at least 6 g of fiber per 100 g may carry the ‘high fiber’ nutrition claim. Therefore, all tested pomace jams meet this criterion. It is well established that dietary fiber intake provides various health benefits, including reducing blood pressure and alleviating the symptoms of diabetes [42].

The insoluble dietary fiber (IDF) content was similar for all tested samples, indicating that the observed differences in total dietary fiber content were primarily due to variations in the soluble dietary fiber (SDF) fraction (Table 3). The highest soluble fiber content was noticed in the apple pomace spreads (3.98–4.28%), followed by the mixed fruit pomace spreads, and the lowest levels in tomato and grape pomace spreads.

Insoluble dietary fiber consists of hemicellulose, cellulose, and lignin, while soluble dietary fiber includes substances such as pectin and other polysaccharides. Soluble dietary fibers are particularly important due to their water solubility, and they have the ability to retain water by forming networks within jams, improving the water retention capacity of the product [43]. Generally, fruits have higher levels of insoluble dietary fiber than soluble dietary fiber [44], and thus, the ratio of IDF/SDF is very important. The apple pomace spreads presented the lowest IDF/SDF ratio (2.36%), similar to the mixed pomaces, followed by the tomato and grape pomace spreads. In some cases, replacing pectin with dietary fibers in commercial strawberry jams has shown no significant differences in quality attributes, while enhancing their sensory attributes [45].

### 3.2. Color Parameters

**Figure 1:**
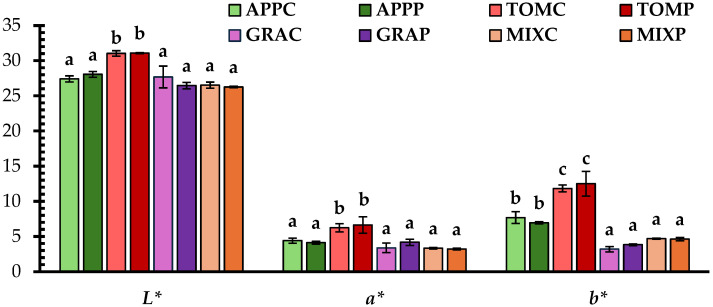
Color parameters of fruit pomace spreads; notation of samples as in Table 1. Mean values with different letters for the same parameter are significantly different (Tukey’s test, *p* < 0.05).

The color parameters L*, a*, and b* were evaluated in fruit pomace spreads (Figure 1). The L* and a* values, representing lightness and redness, respectively, were higher in the tomato pomace spreads, compared to the other samples. For a*, this is likely due to the presence of specific bioactive colorant compounds such as lycopene, which is responsible for the red color in tomatoes [46].

Regarding the b* (yellowness) color parameter, the lowest values were observed in the grape and mixed pomace spreads, followed by the apple and tomato pomace spreads. The grape and mixed pomace spreads exhibited less yellow pigmentation, which could be attributed to their lower carotenoid content compared to the other samples, as the presence of carotenoid pigments is associated with higher b* values [6]. Tomato pomace contains carotenoid pigments, such as lycopene, β-carotene, and γ-carotene [47], which likely contribute to the higher red-yellow pigmentation observed in the tomato pomace spreads.

### 3.3. Rheological Properties of Fruit Pomace Spreads

**Figure 2:**
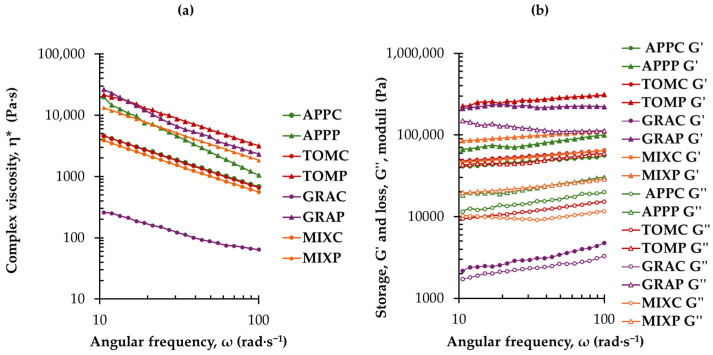
Frequency dependence of (**a**) complex viscosity (η*) and (**b**) storage (G′) and loss (G″) moduli of fruit pomace spreads at 20 °C and 0.1% strain; notation of samples as in Table 1.

Non-Newtonian, pseudoplastic, flow behavior was observed in all tested spreads, as with increasing angular frequency there was a reduction of the complex viscosity (Figure 2a). Interestingly, η* of the GRAC sample had the lowest complex viscosity values and remained relatively more stable over the range of the tested frequencies, implying a rather weak shear-thinning effect. Overall, the mechanical spectra (Figure 2b) revealed a weak gel-like behavior for all tested samples, as G′ was greater than G″. The spread with grape pomace alone (without added pectin) showed the lowest G′ and G″ values and a slight dependence on frequency, whereas the other tested samples exhibited frequency-independent responses for G′ and G″.

Τomato and grape pomace spreads, with the addition of pectin, exhibited the highest G′ values and lowest tanδ values, indicating a more pronounced gel-like behavior (Figure 2b and Table 4). This suggests that the inclusion of pectin enhanced their gelling properties. The tanδ values at the selected angular frequency of 43.80 rad∙s^−1^ (Table 4), were lower than 1, confirming the gel-like behavior of all the tested pomace spreads. Higher tanδ values indicate weaker gel structures, implying lower gel strength. The GRAC sample demonstrated the highest tanδ value (0.76 ± 0.01) and the lowest G′, G″, and η* values (4150 ± 776 Pa, 3153 ± 549 Pa, and 134.6 ± 22.8 Pa∙s, respectively) suggesting a weak gel with a less stable network structure compared to the other samples.

It has been previously postulated that the dietary fiber content affects the thickening of gels, as dietary fibers are known for their water-holding capacity [6,48]. In this study, apple and mixed pomace spreads contained higher TDF levels than grape and tomato pomace spreads. The elevated fiber content in these samples have likely established a sufficiently robust gel matrix, and the addition of pectin contributed slightly further to gel formation. In contrast, in tomato and grape pomace spreads, with lower TDF content, pectin addition resulted in a proportionally greater increase in gel strength (Figure 2 and Table 4). The gelation mechanism of pectin is driven by noncovalent interactions between pectin chains resulting in a network of interconnected three-dimensional structures. For low methoxy pectins, further to hydrophobic contacts and hydrogen bonds between the methyl ester groups of the pectin chains, ionotropic gelation mediated by the divalent Ca^2+^ can occur, stabilizing the junction zones where these interchain associations are formed [49].

### 3.4. Textural Properties of Fruit Pomace Spreads

The highest firmness (F_max_) and spreadability work (SW) values were observed for the mixed pomace spreads followed by samples with grape pomaces, while the tomato pomace spreads with or without the addition of pectin, exhibited the lowest values (Table 4). Although the TOMP sample displayed the highest G′, G″, and η* values, it seems that its resistance to deformation was relatively weak compared to the other spreads (Table 4). This discrepancy in the findings between small and large deformation mechanical testing can be encountered in gels containing high amounts of insoluble particles, such as fruit pomace spreads. Insoluble particles can reinforce the three-dimensional network at the microstructural level by acting as fillers and restricting flow [6]. However, if these particles are poorly interconnected, they become prone to disruption under applied force during large deformation mechanical testing. This leads to interruptions in macrostructural continuity and, consequently, lower resistance during spreading. The firmest and most mechanically resistant macrostructure was observed for MIXP, while the addition of pectin affected only this mixed pomace spread. In a previous study, no differences in firmness were noted among apple pomace jams with the addition of varying concentrations of pectin, xanthan gum, or a combination of both [50]. On the other hand, Mohammadi-Moghaddam et al. [51] reported an increase in firmness as the pectin concentration increased in black plum peel jams.

### 3.5. Total Phenolic Content, Antioxidant Capacity, and Phenolic Compounds of Dried Fruit Pomaces and Pomace Spreads

#### 3.5.1. Total Phenolic Content and Antioxidant Capacity

**Figure 3:**
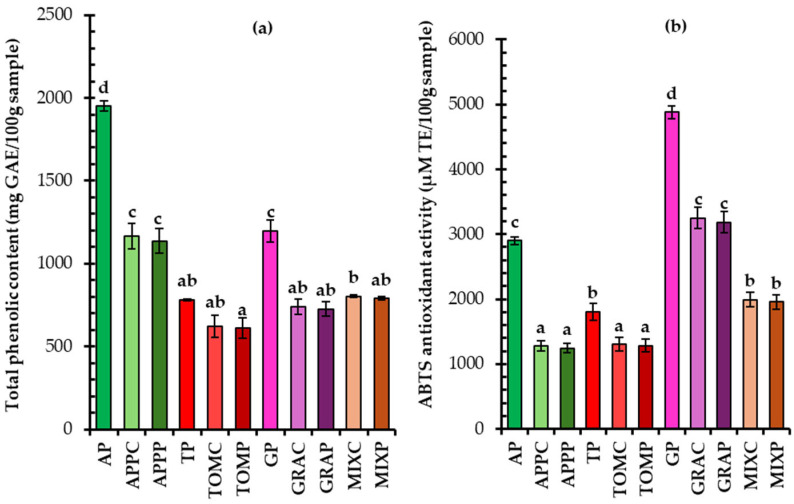
Total phenolic content (**a**) and antioxidant activity (**b**) of fruit pomace spreads; AP: apple pomace, TP: tomato pomace, GP: grape pomace; notation of spread samples as in Table 1; mean values with different letters for the same parameter are significantly different (Tukey’s test, *p* < 0.05).

A higher total phenolic content (TPC) was found in the apple pomace (AP), followed by the grape (GP) and tomato pomace (TP) (Figure 3a). The findings also indicate that fruit pomace spreads can be a rich source of phenolic compounds, despite potential losses occurring during fruit juice processing. Comparable, lower or even higher TPC values have been reported for apple, tomato, and grape pomaces in previous studies [19,20,50,52]. Such variations may be attributed to differences in raw material composition (variety, agronomic conditions, fruit maturity, etc.), processing methods and storage conditions applied to the fruit by-products (waste management).

Among the pomace spreads, apple pomace spreads exhibited the highest TPC values, as expected (Figure 3a). MIX spreads (a mixture of apple and grape pomaces) presented lower TPC values than those made from apple pomace, with values closer to those of the grape pomace spreads. This could be attributed to differences in the sensitivity of various phenolic compounds in the pomaces to thermal treatment during spread production, which depends on their chemical structure and the fruit’s origin and variety, as well as the temperature and duration of processing. During thermal processing, oxidation, degradation, and polymerization reactions may occur, along with the decomposition of polymeric phenolic compounds and the breakdown of polyphenols into simpler phenolic molecules. These changes can result in either a decrease or an increase in the total phenolic content and antioxidant capacity of the final product [53]. In this study, thermal treatment resulted in a reduction of the total phenolic content across all fruit pomace-based spreads, compared to the respective dried pomaces. The apple pomace spread showed a 41% decrease, the grape pomace spread 39%, the tomato pomace spread 21%, and the mixed pomace spread 49%.

Interestingly, in terms of ABTS antioxidant activity, the grape pomace exhibited the highest antioxidant potential (Figure 3b). As a result, grape pomace spreads also showed the highest antioxidant activity among all the spreads, followed by the MIX spreads, which, as expected, had intermediate values between those of the grape and apple pomace spreads. Tomato and apple pomace spreads displayed similar antioxidant activity levels. Furthermore, regardless of fruit origin, a decrease in the antioxidant activity of the pomaces was noted during spread production due to thermal processing.

#### 3.5.2. Phenolic Compounds

**Figure 4:**
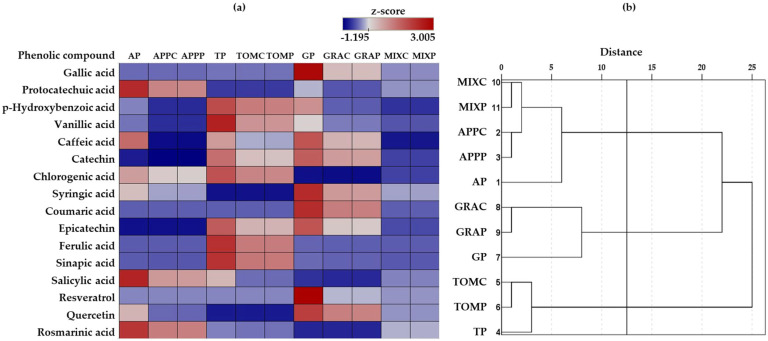
Heatmap (**a**) and dendrogram obtained using Euclidean Distance (**b**) based on the relative concentration change (z-score) of the individual phenolic compounds; AP: apple pomace, TP: tomato pomace, GP: grape pomace, while notation of spread samples as in Table 1.

Phenolic compounds were found in higher concentrations in the dried pomace samples compared to the respective pomace spreads (Table 5). This decrease is likely attributed to the thermal processing applied during spread preparation. The differences in phenolic compound content among the samples are further illustrated by a heatmap (Figure 4a). Catechin and epicatechin were present in higher amounts in both tomato and grape pomace and their respective spreads. The most abundant phenolic compounds identified in the dried tomato pomace and its spread were sinapic acid, p-hydroxybenzoic acid and ferulic acid followed by epicatechin and catechin (Table 5 and Figure 4a). This contrasts with the findings of Bao et al. [19] who reported gallic acid and chlorogenic acid as the predominant phenolic acids of tomato pomace. In grape pomace and its respective spreads, the major phenolic compounds were quercetin, gallic acid, and epicatechin, followed by p-hydroxybenzoic acid and catechin (Table 5 and Figure 4a), consistent with results from a previous study [54]. In the present study, the most abundant phenolics in apple pomace and its spreads were protocatechuic acid, quercetin, and salicylic acid, as described elsewhere [55,56], followed by rosmarinic acid and p-hydroxybenzoic acid (Table 5 and Figure 4a). Variations in phenolic compound content could be ascribed to differences in fruit processing conditions, the type of pomace obtained, and the phenolic extraction method employed for their determination.

Moreover, to investigate similarities in phenolic profiles among the samples, hierarchical cluster analysis was performed using z-scores standardized data (Figure 4b). The samples MIXC, MIXP, APPC, APPP, and AP clustered together, suggesting a similar phenolic composition. This observation was expected, as mixed pomace spreads are formulated with the inclusion of dried apple pomace. Notably, the mixed pomace samples displayed dissimilarity with grape pomace spread samples and exhibited a closer resemblance to apple pomace samples than to grape pomace samples; this may suggest a relatively more extensive destruction and/or transformation of some phenolic compounds in grape pomace than in apple pomace during spread production.

### 3.6. Sensory Attributes

**Figure 5:**
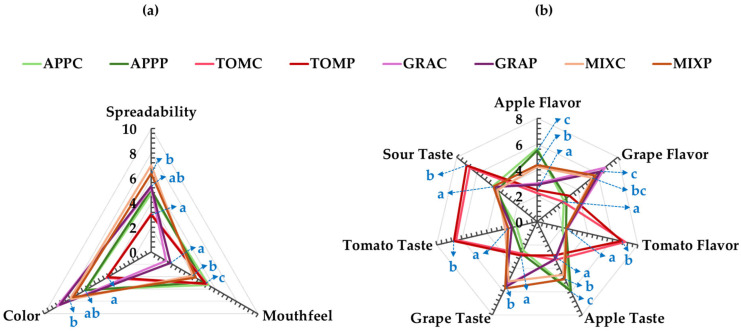
Sensory analysis of fruit pomace spreads: (**a**) evaluation of spreadability, mouthfeel, and color; (**b**) flavor and taste attributes of apple, grape, and tomato; notation of samples as in Table 1. Mean values with different letters for the same parameter are significantly different (Tukey’s test, *p* < 0.05).

The samples MIXC and MIXP received the highest spreadability scores (Figure 5a), indicating that the assessors found it difficult to spread them on a rusk. In contrast, tomato pomace spreads, with or without the addition of pectin, received the lowest scores, highlighting the easy spreading of these samples. These findings align with the results from the instrumental analysis using the texture analyzer (Table 4).

In terms of mouthfeel perception (Figure 5a), the samples containing grape pomace received the lowest scores, indicating products with a granular texture, which was undesirable for the assessors. Interestingly, the incorporation of apple pomace (MIX samples), mediates this coarse sensation. Apple pomace spreads and TOMP products were found to have the smoothest texture, suggesting a more pleasant mouthfeel.

The darkest color (Figure 5a) was noted in grape pomace samples (individually or when mixed with apple), while a lighter color was perceived in tomato pomace spreads, in agreement with the chromameter measurements (Figure 1). Additionally, the spreads seemed to preserve the distinct aroma and taste (Figure 5b) corresponding to the fruits from which the pomaces originated. Notably, the MIX sample preserved both apple and grape aroma and taste, which the assessors were able to identify, recognizing the fruit sources used in the formulation. All the tested samples had a noticeably sour taste, with tomato pomace spreads showing the most intense sourness. This attribute could be attributed to the higher amounts of citric acid added to these spread products for lowering the pH value of the pomaces.

**Figure 6:**
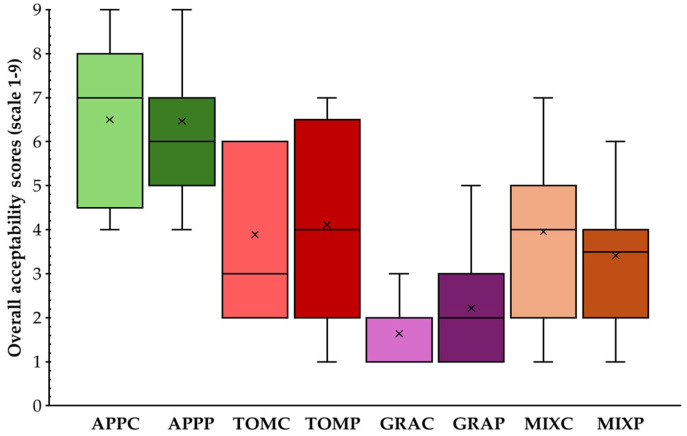
Overall acceptability scores of fruit pomace spreads; the interquartile range (25th to 75th percentile) of the actual values is represented by the boxes, the highest values by the upper whiskers, the lowest values by the lower whiskers, the median values by the horizontal line (-) and mean values by ‘x’; notation of samples as in Table 1.

Overall consumer acceptance scores are presented in Figure 6. The highest mean scores were given to the apple pomace spreads, with the APPP sample also receiving scores with lower variability, indicating greater homogeneity in the consumers preferences and suggesting that this product would be most widely preferred. Hussein et al. [57] also observed higher overall acceptability scores for apple pomace spreads compared to carrot, banana, and mandarin by-products. The lowest scores for grape pomace spreads were likely attributed to the granular texture noted by the assessors.

### 3.7. Principal Component Analysis

**Figure 7:**
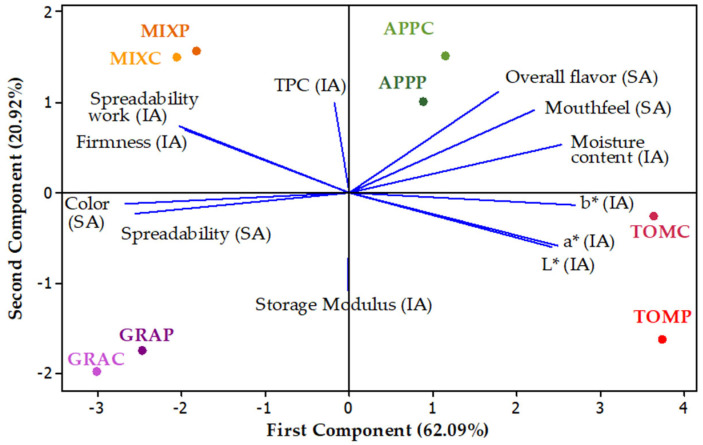
Principal component analysis of fruit pomace spreads. IA, Instrumental Analysis results; SA, Sensory Analysis results; notation of samples as in Table 1.

PCA was performed to explore the relationships between the instrumental and sensory parameters (Figure 7). The PCA biplot was generated based on the first two principal components. Eigenvalues, explained variance, cumulative variance, and variable loadings from principal component analysis results are presented in Table S1. First and second components accounted for ~83.01% of the total variance. The addition of pectin did not seem to affect the overall sample clustering. Spreads containing pectin showed a strong correlation with the samples without the addition of pectin for pomaces of the same plant origin, suggesting that pectin addition did not alter the major underlying characteristics of each type of spread sample. Interestingly, the MIX spread (a combination of apple and grape pomace), aligned closely with grape pomace spreads along the first component and with apple pomace spreads along the second component.

The overall flavor, mouthfeel, and moisture content were strongly correlated to each other and showed a negative correlation with color intensity and spreadability scores, based on the sensory analysis results (Figure 7). These findings indicate that the overall flavor perception, defined as the general sensory impression experienced by the assessors, is associated with spreads exhibiting a smooth texture, an absence of grittiness, high moisture content, and a light color. Furthermore, the ease of spreadability on a rusk also contributed to higher sensory acceptance scores. As expected, the sensory perception based on the difficulty in spreading the products on a rusk was positively correlated with the spreadability work and firmness values obtained through an instrumental analysis of the fruit pomace spreads. Similarly, the perceived color intensity (darkness) by the sensory panelists was negatively correlated with the L* values given by colorimeter.

It is noteworthy that spreads derived from different fruit pomaces were grouped into distinct quadrants, reflecting their unique quality characteristics. Thus, PCA confirmed that spreads made from grape pomace, either alone or in combination with apple pomace, were associated with greater difficulty in spreading, as indicated by the small angles between the vectors of all spreadability-related variables (F_max_, SW, and sensory spreadability). In contrast, spreads made solely from apple pomace were linked to a pleasant mouthfeel and intense flavor. These findings are also reflected in the overall acceptability scores given by the panelists, with apple pomace-based spreads receiving the highest scores and grape pomace spreads the lowest.

## 4. Conclusions

In this study, apple, tomato, and grape pomaces, as well as a mixed pomace preparation from apple and grape, were used to make jam-like spreads in an effort to valorize these bioactive by-products of the fruit juice industry. The addition of pectin was also explored to examine its effect on the quality characteristics of the spreads. All spread formulations qualify for the ‘high fiber’ nutrition claim. The insoluble dietary fiber content was similar across all samples, indicating that variations in TDF were primarily due to differences in soluble fiber content; apple pomace spreads had the highest SDF content. The color analysis revealed that tomato pomace spreads exhibited higher L* and a* values among the tested formulations. All spreads demonstrated non-Newtonian, pseudoplastic flow behavior, and weak gel-like behavior (G′ > G″). The texture analysis indicated that pectin addition had a noticeable effect only in the mixed pomace spreads. In contrast, the viscosity and viscoelastic properties of spreads from both tomato and grape pomaces were enhanced by pectin inclusion into the product formulation. In terms of bioactive compounds present in raw materials or final products, the apple pomace exhibited the highest total phenolic content (TPC) in comparison to all other pomaces and, as a result, apple pomace spreads had the highest TPC among all fruit pomace-based spreads. Moreover, the grape pomace presented the highest antioxidant activity. The hierarchical cluster analysis showed that the samples MIXC, MIXP, APPC, APPP, and AP clustered together, suggesting similar phenolic composition profiles. The principal component analysis also indicated that pomace products from different fruit origins can be grouped based on their distinct quality characteristics. A strong correlation was found among flavor intensity, perception of appealing mouthfeel (smooth texture) and moisture content, with these attributes exhibiting a negative correlation with color intensity and difficulty in spreading.

In conclusion, apple, tomato, and grape pomaces, individually or in combination, can be effectively utilized to produce fruit-based spreads, with or without the addition of pectin. Among the formulations tested, the apple pomace spread with the addition of pectin was the most favorably perceived by consumers, as they appreciated its pleasant mouthfeel and ease of spreading. Overall, fruit industry by-products can be valorized to prepare fruit pomace spreads with high dietary fiber and phenolic compound contents, having acceptable sensory attributes, instead of being discarded as waste materials.

## Figures and Tables

**Table 1:** Composition of ingredients in making the fruit pomace spreads.

Sample	Abbreviation	Pomace (g)	Sugar (g)	Citric Acid (g)	Pectin (g)	CaCl_2_ (g)	Water (g)
Apple pomace spread	APPC	100.00	100.00	-	-	-	400.00
	APPP	100.00	100.00	-	5.00	5.00	400.00
Tomato pomace spread	TOMC	100.00	100.00	2.85	-	-	400.00
	TOMP	100.00	100.00	2.85	5.00	5.00	400.00
Grape pomace spread	GRAC	100.00	100.00	1.60	-	-	300.00
	GRAP	100.00	100.00	1.60	5.00	5.00	300.00
Apple–grape (1:1) pomace spreads	MIXC	100.00	100.00	-	-	-	400.00
	MIXP	100.00	100.00	-	5.00	5.00	400.00

**Table 2:** pH values, water activity (a_w_), total titratable acidity and moisture content of fruit pomace spreads.

Sample ^1^	pH	Titratable Acidity (Expressed as g of Citric Acid/100 g Spread)	a_w_	Moisture (%)
APPC	3.22 ± 0.06ab ^2^	1.36 ± 0.03c	0.94 ± 0.01c	58.90 ± 3.65d
APPP	3.30 ± 0.00b	1.19 ± 0.08bc	0.95 ± 0.00c	62.43 ± 0.55d
TOMC	3.36 ± 0.12b	1.15 ± 0.58bc	0.96 ± 0.02c	65.86 ± 0.86d
TOMP	3.34 ± 0.06b	1.06 ± 0.16abc	0.97 ± 0.00c	62.99 ± 1.99d
GRAC	3.34 ± 0.11b	1.18 ± 0.03bc	0.88 ± 0.03a	36.14 ± 3.14a
GRAP	3.29 ± 0.01b	0.51 ± 0.13a	0.90 ± 0.00ab	38.97 ± 6.46ab
MIXC	3.42 ± 0.02b	0.66 ± 0.03ab	0.91 ± 0.01b	47.77 ± 1.47bc
MIXP	3.05 ± 0.08a	0.60 ± 0.06ab	0.90 ± 0.0ab	49.23 ± 0.25c

^1^ Notation of samples as in Table 1. ^2^ Mean values with different letters for the same parameter are significantly different (Tukey’s test, *p* < 0.05).

**Table 3:** Protein and dietary fiber contents of fruit pomace spreads.

Sample ^1^	Protein (%)	SDF (%) ^2^	IDF (%) ^2^	TDF (%) ^2^	IDF/SDF ^2^
APPC	1.52 ± 0.12a ^3^	4.28 ± 0.42c	9.85 ± 0.87a	14.13 ± 0.44e	2.36 ± 0.44a
APPP	1.55 ± 0.14a	3.98 ± 0.48c	9.16 ± 0.99a	13.14 ± 0.51de	2.36 ± 0.53a
TOMC	3.21 ± 0.24bc	2.01 ± 0.03a	8.10 ± 0.17a	10.11 ± 0.13a	4.03 ± 0.15bc
TOMP	3.13 ± 0.28b	1.96 ± 0.04a	7.89 ± 0.20a	9.85 ± 0.16a	4.03 ± 0.18bc
GRAC	3.96 ± 0.23d	1.99 ± 0.07a	9.00 ± 0.87a	10.99 ± 0.80abc	4.55 ± 0.60c
GRAP	3.76 ± 0.27cd	1.89 ± 0.08a	8.54 ± 1.01a	10.42 ± 0.93ab	4.55 ± 0.73c
MIXC	2.81 ± 0.05b	3.14 ± 0.18b	9.42 ± 0.00a	12.56 ± 0.18cde	3.02 ± 0.17ab
MIXP	2.65 ± 0.06b	2.94 ± 0.20b	8.85 ± 0.01a	11.78 ± 0.21bcd	3.03 ± 0.20ab

^1^ Notation of samples as in Table 1. ^2^ SDF, Soluble dietary fiber; IDF, Insoluble dietary fiber; TDF Total dietary fiber; IDF/SDF Insoluble/Soluble dietary fiber. ^3^ Mean values with different letters for the same parameter are significantly different (Tukey’s test, *p* < 0.05).

**Table 4:** Rheological parameters at angular frequency 43.8 rad∙s^−1^ (0.1 strain, 20 °C) and textural properties (large deformation mechanical test) of fruit pomace spreads.

Sample ^1^	Storage Modulus, G′ (Pa)	Loss Modulus, G″ (Pa)	Complex Viscosity, η* (Pa∙s)	Damping Factor, tanδ	Firmness, F_max_ (N)	Spreadability Work, SW (N∙mm)
APPC	50,000 ± 6124ab ^2^	16,015 ± 2870ab	1385.9 ± 306.9abc	0.32 ± 0.02b	59.22 ± 2.05b	59.23 ± 2.66b
APPP	73,900 ± 6777abc	22,300 ± 1633ab	3270.0 ± 253.1bc	0.30 ± 0.01b	58.85 ± 2.00b	49.07 ± 10.29b
TOMC	57,225 ± 9941abc	12,560 ± 2400a	1343.0 ± 234.3abc	0.23 ± 0.08ab	5.40 ± 0.69a	4.53 ± 0.80a
TOMP	279,600 ± 61,564d	51,178 ± 12,021c	6502.5 ± 1435.0d	0.18 ± 0.00a	8.89 ± 0.13a	7.51 ± 0.69a
GRAC	4150 ± 776a	3153 ± 549a	134.6 ± 22.8a	0.76 ± 0.01c	151.69 ± 2.85c	106.82 ± 2.42c
GRAP	168,375 ± 41,335cd	46,225 ± 8798c	3933.3 ± 936.3cd	0.28 ± 0.02ab	163.19 ± 49.71c	91.43 ± 7.45c
MIXC	47,233 ± 6002ab	13,027 ± 2957a	1122.7 ± 154.2ab	0.27 ± 0.03ab	384.57 ± 15.33d	214.71 ± 11.96d
MIXP	160,333 ± 55,769bc	36,700 ± 10,044bc	3760.0 ± 1288.2bc	0.23 ± 0.02ab	440.86 ± 14.97e	255.60 ± 13.72e

^1^ Notation of samples as in Table 1. ^2^ Mean values with different letters for the same parameter are significantly different (Tukey’s test, *p* < 0.05).

**Table 5:** Individual phenolic compounds (µg/g sample) in fruit pomaces and fruit pomace spreads.

Phenolic Compound	AP ^1^	APPC	APPP	TP	TOMC	TOMP	GP	GRAC	GRAP	MIXC	MIXP
Gallic acid	n.d. ^2^	n.d.	n.d.	1.95 ± 1.09a	1.63 ± 0.06a	1.61 ± 0.04a	123.59 ± 2.92d	31.05 ± 1.60c	30.44 ± 1.28c	8.31 ± 0.74b	8.18 ± 0.73b
Protocatechuic acid	337.60 ± 12.58f ^3^	196.41 ± 0.34e	191.62 ± 0.27e	n.d.	n.d.	n.d.	68.16 ± 3.32d	18.47 ± 0.30b	18.11 ± 0.25b	52.19 ± 4.30c	51.34 ± 3.46c
p-Hydroxybenzoic acid	41.10 ± 0.98b	20.93 ± 1.75a	20.42 ± 1.40a	129.95 ± 6.90e	102.65 ± 2.12d	100.98 ± 1.70d	92.47 ± 4.61c	33.78 ± 0.74b	33.12 ± 0.60b	23.05 ± 0.71a	22.68 ± 0.57a
Vanillic acid	7.23 ± 0.58b	0.48 ± 0.22a	0.46 ± 0.18a	56.07 ± 4.43e	28.92 ± 1.34d	28.44 ± 1.08d	16.54 ± 0.79c	8.04 ± 0.39b	7.88 ± 0.31b	4.44 ± 0.22ab	4.37 ± 0.18ab
Caffeic acid	1.91 ± 0.26de	0.49 ± 0.07a	0.48 ± 0.07a	1.56 ± 0.16cd	1.02 ± 0.02abc	1.00 ± 0.02ab	2.08 ± 0.20e	1.40 ± 0.30bcd	1.37 ± 0.24bcd	0.54 ± 0.17a	0.54 ± 0.14a
Catechin	8.60 ± 1.53ab	1.05 ± 0.09a	1.02 ± 0.12a	83.99 ± 1.49e	51.04 ± 3.93c	50.20 ± 3.16c	91.54 ± 2.66e	64.60 ± 4.28d	63.34 ± 3.42d	17.08 ± 6.12b	16.49 ± 4.59b
Chlorogenic acid	27.51 ± 1.92d	19.93 ± 0.64c	19.45 ± 0.51c	41.86 ± 0.59f	32.08 ± 0.60e	31.56 ± 0.18e	1.85 ± 0.50a	1.32 ± 0.13a	1.35 ± 0.04a	6.89 ± 0.95b	6.78 ± 0.76b
Syringic acid	28.09 ± 1.33b	16.84 ± 0.95a	16.42 ± 0.76a	n.d.	n.d.	n.d.	67.80 ± 3.68d	37.10 ± 3.90c	36.38 ± 3.13c	16.85 ± 0.85a	16.57 ± 0.68a
Coumaric acid	n.d.	n.d.	n.d.	n.d.	n.d.	n.d.	0.68 ± 0.16a	0.41 ± 0.22a	0.40 ± 0.017a	n.d.	n.d.
Epicatechin	n.d.	n.d.	n.d.	98.85 ± 3.69c	57.01 ± 7.27b	56.08 ± 5.84b	102.54 ± 6.75c	47.89 ± 1.09b	46.95 ± 0.87b	14.78 ± 3.60a	14.53 ± 2.89a
Ferulic acid	n.d.	n.d.	n.d.	110.68 ± 0.30d	69.11 ± 0.47c	67.98 ± 0.38c	1.90 ± 0.45b	0.74 ± 0.05a	0.72 ± 0.04a	0.22 ± 0.03a	0.22 ± 0.02a
Sinapic acid	3.36 ± 1.07a	1.25 ± 0.08a	1.22 ± 0.06a	250.71 ± 4.97c	164.07 ± 6.99b	161.39 ± 5.61b	10.75 ± 1.41a	6.85 ± 0.84a	7.00 ± 0.88a	2.22 ± 0.35a	2.18 ± 0.28a
Salicylic acid	92.06 ± 1.47e	47.37 ± 2.61d	46.21 ± 2.08d	37.05 ± 3.19c	12.59 ± 1.40b	12.38 ± 1.12b	3.18 ± 0.32a	1.54 ± 0.55a	1.51 ± 0.44a	15.71 ± 0.82b	15.46 ± 0.66b
Resveratrol	n.d.	n.d.	n.d.	n.d.	n.d.	n.d.	6.15 ± 0.64b	0.39 ± 0.05a	0.38 ± 0.04a	0.11 ± 0.02a	0.11 ± 0.02a
Quercetin	94.40 ± 0.76c	31.21 ± 0.78a	30.45 ± 0.63a	n.d.	n.d.	n.d.	197.17 ± 12.07e	138.08 ± 2.45d	135.38 ± 1.97d	45.77 ± 3.12b	45.02 ± 2.50b
Rosmarinic acid	59.86 ± 4.47d	40.30 ± 0.16c	39.32 ± 0.13c	10.22 ± 1.13a	8.89 ± 0.45a	8.75 ± 0.37a	n.d.	n.d.	n.d.	14.60 ± 0.42b	14.36 ± 0.34b

^1^ AP: apple pomace, TP: tomato pomace, GP: grape pomace, while notation of spread samples as in Table 1. ^2^ n.d.: not detected. ^3^ Mean values with different letters for the same phenolic compound are significantly different (Tukey’s test, *p* < 0.05).

## Data Availability

The original contributions presented in the study are included in the article/Supplementary Materials, further inquiries can be directed to the corresponding author.

## Supplementary Materials

The following supporting information can be downloaded at: https://www.mdpi.com/article/10.3390/foods14132224/s1, Table S1: Eigenvalues, explained variance, cumulative variance, and variable loadings from principal component analysis results.
