# Supplementary material for: Physicochemical and Sensory Evaluation of Spreads Derived from Fruit Processing By-Products
Source: Foods. 2025 Jun 24;14(13):2224. doi: 10.3390/foods14132224 (PMC12249053; doi:10.3390/foods14132224)
Supplement: Supplementary file 1 [file foods-14-02224-s001.zip › foods-3695767-supplementary.pdf]

Table S1. Eigenvalues, explained variance, cumulative variance, and variable loadings from principal component analysis results.

Variable	PC1	PC2	PC3	PC4	PC5	PC6	PC7	PC8	PC9	PC10	PC11	PC12
Eigenvalue	7.4504	2.5104	1.4137	0.5041	0.0847	0.0300	0.0067	0.0000	0.0000	0.0000	0.0000	0.0000
Explained Variance (%)	62.10	20.90	11.80	4.20	0.70	0.20	0.10	0.00	0.00	0.00	0.00	0.00
Cumulative Variance (%)	62.10	83.00	94.80	99.00	99.70	99.90	100.00	100.00	100.00	100.00	100.00	100.00
Loadings												
L* (IA)	0.324	-0.238	0.138	-0.139	0.616	-0.489	0.212	-0.182	-0.216	0.229	0.015	-0.067
a* (IA)	0.333	-0.233	0.098	0.053	-0.471	0.205	-0.155	-0.164	-0.476	0.523	-0.082	-0.026
b* (IA)	0.361	-0.052	0.111	0.080	-0.043	-0.160	-0.234	0.743	-0.244	-0.281	0.253	-0.107
Spreadability (SA)	-0.344	-0.092	-0.239	-0.161	0.212	0.018	-0.400	-0.000	-0.467	-0.212	-0.497	-0.272
Color (SA)	-0.361	-0.046	-0.107	-0.088	-0.187	-0.108	0.410	-0.112	-0.567	-0.236	0.457	0.186
Mouthfeel (SA)	0.296	0.362	0.003	0.179	0.086	0.039	-0.401	-0.553	-0.099	-0.320	0.359	-0.180
Storage modulus (IA)	-0.003	-0.429	-0.390	0.799	0.119	0.019	0.069	0.034	0.028	-0.061	-0.004	0.018
Firmness (IA)	-0.262	0.275	0.404	0.343	0.069	0.107	0.286	0.099	-0.116	0.181	0.015	-0.647
Spreadability work (IA)	-0.274	0.293	0.342	0.335	0.209	-0.119	-0.310	0.081	-0.190	0.224	-0.050	0.601
Moisture content (IA)	0.340	0.214	-0.037	-0.005	0.331	0.639	0.359	0.087	-0.238	-0.171	-0.210	0.230
Overall flavor (SA)	0.239	0.444	-0.180	0.178	-0.335	-0.495	0.267	-0.033	-0.091	-0.132	-0.473	0.038
TPC (IA)	-0.023	0.395	-0.651	-0.072	0.162	0.006	-0.062	0.208	-0.044	0.508	0.271	-0.088
